# Re-sensitization of *Mycobacterium smegmatis* to Rifampicin Using CRISPR Interference Demonstrates Its Utility for the Study of Non-essential Drug Resistance Traits

**DOI:** 10.3389/fmicb.2020.619427

**Published:** 2021-02-01

**Authors:** Valwynne Faulkner, Adrienne Adele Cox, Shan Goh, Annelies van Bohemen, Amanda J. Gibson, Oliver Liebster, Brendan W. Wren, Sam Willcocks, Sharon L. Kendall

**Affiliations:** ^1^Pathobiology and Population Sciences, The Royal Veterinary College, Hatfield, United Kingdom; ^2^Institute of Infection, Veterinary and Ecological Sciences, University of Liverpool, Liverpool, United Kingdom; ^3^Department of Clinical, Pharmaceutical & Biological Science, School of Life & Medical Sciences, University of Hertfordshire, Hatfield, United Kingdom; ^4^Centre of Excellence for Bovine TB, Aberystwyth University, Aberystwyth, United Kingdom; ^5^Department of Infection Biology, The London School of Hygiene and Tropical Medicine, London, United Kingdom

**Keywords:** antibiotic resistance, tuberculosis, CRISPRi, mycobacteria, rifampicin resistance

## Abstract

A greater understanding of the genes involved in antibiotic resistance in *Mycobacterium tuberculosis (Mtb)* is necessary for the design of improved therapies. Clustered regularly interspaced short palindromic repeat interference (CRISPRi) has been previously utilized in mycobacteria to identify novel drug targets by the demonstration of gene essentiality. The work presented here shows that it can also be usefully applied to the study of non-essential genes involved in antibiotic resistance. The expression of an ADP-ribosyltransferase (Arr) involved in rifampicin resistance in *Mycobacterium smegmatis* was silenced using CRISPRi and the impact on rifampicin susceptibility was measured. Gene silencing resulted in a decrease in the minimum inhibitory concentration (MIC) similar to that previously reported in an *arr* deletion mutant. There is contradictory evidence for the toxicity of *Streptococcus pyogenes* dCas9 (dCas9_Spy_) in the literature. In this study the expression of dCas9_Spy_ in *M. smegmatis* showed no impact on viability. Silencing was achieved with concentrations of the aTc inducer lower than previously described and with shorter induction times. Finally, designing small guide RNAs (sgRNAs) that target transcription initiation, or the early stages of elongation had the most impact on rifampicin susceptibility. This study demonstrates that CRISPRi based gene silencing can be as impactful as gene deletion for the study of non-essential genes and further contributes to the knowledge on the design and induction of sgRNAs for CRISPRi. This approach can be applied to other non-essential antimicrobial resistance genes such as drug efflux pumps.

## Introduction

Human pulmonary tuberculosis (TB) caused by *Mtb* remains one of the most important infectious diseases in public health worldwide. It is estimated that up to 1.5 million people die of TB annually, making it the highest cause of death from an infectious agent (Global tuberculosis report, 2020). Current TB treatments involve long regimens with four antibiotics: rifampicin, isoniazid, ethambutol and pyrazinamide and have associated side-effects. The length of the treatment and frequent patient non-compliance encourage the occurrence of multi-drug resistant (MDR) and extremely drug resistant (XDR) TB with the emergence of drug resistance in *Mtb* being a considerable public health crisis hindering control and eradication programs (Dheda et al., 2017). The discovery of new drugs and novel targets as well as efforts to improve the efficacy of current available drugs are crucial to control the global epidemic.

Essential genes are attractive novel drug targets, and the demonstration of essentiality can require modulating the expression of the putative essential gene in a merodiploid strain, an approach that is time consuming, costly, and inefficient in organisms such as mycobacteria that are particularly challenging to genetically modify due to low frequencies of recombination and slow growth (Choudhary et al., 2016). The CRISPRi system overcomes a number of these challenges. A catalytically inactive dCas9 protein is expressed and, guided by sgRNAs, targets a specific gene sequence. The resulting complex binds to the target sequence and sterically inhibits transcription initiation or elongation (Larson et al., 2013). The CRISPRi/dCas9 system has been utilized to study essential genes in a number of bacteria including; *Escherichia coli* (Qi et al., 2013), *Streptococcus pneumoniae* (Bikard et al., 2013), *Streptomyces coelicolor* (Tong et al., 2015), *Bacillus subtilis* (Peters et al., 2016), *Clostridium acetobutylicum* (Li et al., 2016), *Corynebacterium glutamicum* (Cleto et al., 2016), *Staphylococcus aureus* (Zhao et al., 2017) and *Pseudomonas* spp. (Tan et al., 2018).

Several papers have described the use of CRISPRi for silencing of essential genes in mycobacteria. Choudhary et al. (2015) introduced the CRISPRi approach in *M. smegmatis, Mycobacterium bovis* BCG Pasteur and *Mtb* using a codon-optimized *S. pyogenes* dCas9_Spy_. Singh et al. (2016) followed with a similar CRISPRi/dCas9 system for use in *Mtb*, in this case a two-plasmid system with dCas9_Spy_ cloned into an integrative plasmid under an anhydro-tetracycline (aTc) inducible promoter, co-expressed with the sgRNA on a second plasmid. Additionally, a single plasmid system using dCas9 derived from *Streptococcus thermophilus* (dCas9_Sth1_) has also been developed and used to silence several putative essential genes allowing the verification of their suitability as drug targets (Rock et al., 2017; McNeil and Cook, 2019).

The majority of previous studies have utilized this system to silence the expression of putative essential drug-targets to demonstrate their requirement for bacterial growth by measuring the impact on the growth of the silenced strain. An alternative approach in the search for new therapeutic strategies is to make the current antibiotics more effective by targeting genes that are not essential for growth but are involved in antibiotic resistance, i.e., re-sensitizing antibiotic resistant strains. This approach has recently been used in *E. coli* where sequence-specific targeting an antibiotic efflux pump using peptide conjugated oligomers not only restored antibiotic sensitivity in resistant strains, but rendered strains hyper-sensitive allowing 2- to 40-fold lower antibiotic doses (Ayhan et al., 2016). Given the time and costs associated with the development of new drugs, the improvement of the efficacy of the existing antimicrobials is a useful alternative strategy. The silencing approach also holds promise as a way to understand bacterial mechanisms of resistance.

In this study, we demonstrate the utilization of the CRISPRi system to restore sensitivity to the antibiotic rifampicin in *M. smegmatis*, a saprophytic species that is intrinsically resistant to rifampicin due the presence of Arr, which inactivates rifampicin by ribosylation (Dabbs et al., 1995). We measure the impact of repression of the *arr* gene on sensitivity to rifampicin by measuring the MIC in silenced strains. In addition, we measure the effect of varying the concentration of the aTc inducer and the position of the sgRNA in relation to the translational start site on the MIC of rifampicin.

## Materials and Methods

### Bacterial Growth and Culture Conditions

*M. smegmatis* strains were grown in Middlebrook 7H9 broth supplemented with 0.2% glycerol, 0.05% Tween 80 (Sigma) and 10% albumin-dextrose-catalase (BD Diagnostics) at 37°C on a shaking platform or on Middlebrook 7H11 agar supplemented with 10% oleic-albumin-dextrose-catalase (BD Diagnostics) at 37°C for 3 days. For plasmid selection, antibiotics were used at the following concentrations: kanamycin 25 μg/ml (Sigma), hygromycin 50 μg/ml (Roche) and aTc 2-200 ng/ml (Sigma). Bacterial growth was measured by monitoring the OD_600_ over time and viability measurements were made using the Miles and Misra technique; spotting 20 μl of a series of 10-fold dilutions of culture onto agar (Miles et al., 1938).

### Construction of sgRNA Expression Plasmids and Strains

The strains and plasmids used in this study are given in Table 1. pRH2502, an integrative plasmid expressing dCas9_Spy_ and pRH2521 expressing the sgRNA scaffold, both from Tet-regulated promoters were acquired from Singh et al. (2016) currently available on Addgene. sgRNAs targeting *arr* were designed using a published protocol (Larson et al., 2013). Protospacer adjacent motif (PAM) sites, “NGG,” upstream and downstream of the annotated translational start site were chosen:- 20 nucleotides downstream of each PAM were selected as a potential genome specific sgRNA. The probability of complementarity to any other region of the genome was analyzed using a basic local alignment search tool (BLAST) (Altschul et al., 1990). Sequences with full length matches specific to *arr* were taken forward. A full length transcribed sgRNA including the terminators and dCas9 handle was designed and M-fold was used to predict the secondary structure of the full length sgRNA transcript (Zuker, 2003). Candidates were selected which were predicted to fold into the dCas9_Spy_ and terminator hairpin loops. Complementary forward and reverse primers using the 20 nucleotide sequence (without the PAM) with appropriate ends for ligation to the pRH2521 vector were designed. All forward primers had “ggga” and all reverse primers had “aaac” added to the 5′ end. Table 2 shows the list of oligonucleotides used to construct the sgRNA plasmids and the strand targeted by each oligonucleotide. Oligos were annealed and cloned into CRISRPi plasmids using BbsI (NEB) as previously described (Choudhary et al., 2015; Singh et al., 2016). One microgram of pRH2502 (dCas9_Spy_ integrative vector) was electroporated at 25 kV, 25 μF with 1,000 Ω resistance into electrocompetent *M. smegmatis* mc^2^155, this strain was grown and further electroporated with 1 μg of pRH2521 expressing the targeted sgRNAs (Parish and Stoker, 1998).

**Table 1:** Strains and plasmids used in this study.

**Strain/plasmid**	**Genotype/Description**	**Source**
**Strains**		
*E. coli* DH5α	*SupE44 ΔlacU169 (lacZΔM15) hsdR17 recA1 endA1 gyrA96 thi-1 relA1*	Invitrogen
*M. smegmatis* mc^2^155	High-frequency transformation mutant ATCC 607	Snapper et al., 1990
Msm_dCas9_	*M. smegmatis* mc^2^155 with integrative plasmid containing *dcas9_*Spy*_*(pRH2502), kan^R^	This study
Msm_dCas9__control	Msm_dCas9_ with non-integrative plasmid for inserting sgRNA (pRH2521). Empty vector control, kan^R^, hyg^R^	This study
Msm_dCas9__arr1	Msm_dCas9_ with plasmid containing sgRNA targeting between−47 bp and −28 bp upstream arr start codon (pSG_arr1) kan^R^, hyg^R^	This study
Msm_dCas9__arr2	Msm_dCas9_ with sgRNA targeting between−27 bp and −8 bp upstream *arr* start codon (pSG_arr2), kan^R^, hyg^R^	This study
Msm_dCas9__arr3	Msm_dCas9_ with sgRNA targeting between +13 bp and +32 bp downstream of the *arr* start codon (pSG_arr3), kan^R^, hyg^R^	This study
**Plasmids**		
pRH2502	Integrative plasmid derived from pTC-0X-1L, expressing dCas9_Spy_ from an inducible tetRO promoter (uv15tetO)	Singh et al., 2016
pRH2521	Non-integrative plasmid derived from pTE-10M-0X, expressing sgRNA from an inducible tetRO promoter (Pmyc1tetO)	Singh et al., 2016
pSG_arr1	sgRNA targeting between −47 bp to −28 bp upstream of the *arr* start codon cloned into pRH2521	This study
pSG_arr2	sgRNA targeting between −27 bp to −8 bp upstream *arr* start codon cloned into pRH2521	This study
pSG_arr3	sgRNA targeting between +13 bp to +32 bp downstream of the *arr* start codon cloned into pRH2521	This study
pSG_oligo2	sgRNA targeting between −50 bp to −38 bp upstream of the *arr* start codon cloned into pRH2521	This study
pSG_oligo3	sgRNA targeting between −32 bp to −20 bp upstream of the *arr* start codon cloned into pRH2521	This study
pSG_oligo4	sgRNA targeting between +66 bp to +78 bp downstream of the *arr* start codon cloned into pRH2521	This study
pSG_oligo5	sgRNA targeting between −74 bp to −56 bp upstream of the *arr* start codon cloned into pRH2521	This study
pSG_oligo6	sgRNA targeting between −56 bp to −38 bp upstream of the *arr* start codon cloned into pRH2521	This study
pSG_oligo7	sgRNA targeting between +60 bp to +78 bp downstream of the *arr* start codon cloned into pRH2521	This study
pSG_oligo8	sgRNA targeting between +166 bp to +184 bp downstream of the *arr* start codon cloned into pRH2521	This study
pSG_oligo9	sgRNA targeting between −53 bp to −35 bp upstream of the *arr* start codon cloned into pRH2521	This study
pSG_oligo10	sgRNA targeting between −27 bp to −9 bp upstream of the *arr* start codon cloned into pRH2521	This study

*The targeting region given does not include the 3 bp PAM site*.

**Table 2:** Primers used in this study.

**Oligonucleotide name**	**Sequence**	**Strand targeted**	**Region targeted relative to *arr* start**
**CRISPRi Oligonucleotides**			
arr1_Forward	GGGACGGAACGCCGATTGTGCACC	Coding strand	−47 bp to −28 bp upstream
arr1_Reverse	AAACGGTGCACAATCGGCGTTCCG		
arr2_Forward	GGGACCCCCTGTTCCGTGTGTGAT	Coding strand	−27 bp to −8 bp upstream
arr2_Reverse	AAACATCACACACGGAACAGGGGG		
arr3_Forward	GGGATGCACTTCGAACGGTTTCGG	Coding strand	+13 bp to +32 bp downstream
arr3_Reverse	AAACCCGAAACCGTTCGAAGTGCA		
Oligo 2_Forward	GGGACCGGGTGCACAAT	Non-coding	−50 bp to −38 bp upstream
Oligo 2_Reverse	AAACATTGTGCACCCGG		
Oligo 3_Forward	GGGATTCCGATCACACA	Non-coding	−32 bp to −20 bp upstream
Oligo 3_Reverse	AAACTGTGTGATCGGAA		
Oligo 4_Forward	GGGAGAGCTCAAGGTGG	Non-coding	+66 bp to +78 bp downstream
Oligo 4_Reverse	AAACCCACCTTGAGCTC		
Oligo 5_Forward	GGGATGGGGCTTGCGGCAAGGCC	Non-coding	−74 bp to −56 bp upstream
Oligo 5_Reverse	AAACGGCCTTGCCGCAAGCCCCA		
Oligo 6_Forward	GGGACCGGTGCCGGGTGCACAAT	Non-coding	−56 bp to −38 bp upstream
Oligo 6_Reverse	AAACATTGTGCACCCGGCACCGG		
Oligo 7_Forward	GGGAAAGGCCGAGCTCAAGGTGG	Non-coding	+60 bp to +78 bp downstream
Oligo 7_Reverse	AAACCCACCTTGAGCTCGGCCTT		
Oligo 8_Forward	GGGAAGTTCGGCACCCCACACCG	Coding	+166 bp to +184 bp downstream
Oligo 8_Reverse	AAACCGGTGTGGGGTGCCGAACT		
Oligo 9_Forward	GGGACCGATTGTGCACCCGGCAC	Coding	−53 bp to −35 bp upstream
Oligo 9_Reverse	AAACGTGCCGGGTGCACAATCGG		
Oligo 10_Forward	GGGACCCCTGTTCCGTGTGTGAT	Coding	−27 bp to −9 bp upstream
Oligo 10_Reverse	AAACATCACACACGGAACAGGGG		
**RT-qPCR primers**			
sigA_forward	CCTGGAACTCGACGACCTC		
sigA_reverse	GACTCTTCCTCGTCCCACAC		
arr_forward	AGATCACCCAGACGTTGGAC		
arr_reverse	CTCGGGCTCGACGATGTAAA		

*These are underlined in the table first four bases in each of the CRISPRi primer sets are used for cloning the sgRNA into pRH2521. The region targeted does not include the 3 bp PAM site*.

### RNA Extraction

Total RNA was extracted as previously described (Rustad et al., 2009). Cultures were grown to OD_600_ 0.1–0.2 and induced with 0, 50 or 200 ng/ml of aTc for an hour, followed by centrifugation at 2,000 x g at 4 °C for 5 min. Pellets were resuspended in TRIzol (Sigma) on ice and added to 1.5 ml tubes containing 0.1 mm glass beads. Cells were disrupted by three cycles of 30 s pulses at speed 6,500 rpm using a Precellys homogenizer. RNA was purified using a Qiagen RNeasy kit combined with on-column DNase digestion according to the manufacturer's instructions. Quantity and quality were determined using a DeNovix Spectrophotometer (DeNovix Inc, USA) and agarose gel electrophoresis.

### RT-qPCR

To remove traces of contaminating DNA, RNA samples were treated with RNase-free DNase I (Invitrogen). cDNA was synthesized from 100 ng of RNA using Superscript III Reverse transcriptase (ThermoFisher) according to manufacturer instructions. In order to quantify the expression of arr, qPCRs were performed using PowerUp SYBR Green Master Mix (ThermoFisher) with 1 μl of cDNA and 0.3 μM of either sigA primers or arr primers (Table 2) in a final volume of 20 μl. Samples were run on a BioRad CFX96 analyser at 50 °C for 2 min, 95 °C for 2 min, followed by 39 cycles of 95 °C for 15 s, 60 °C for 1 min and 75 °C for 30 s. The mRNA levels of *arr* were normalized against the mRNA of the reference gene *sigA*. Three biological replicates were run, with technical duplicates. Melting curve analysis was carried out to verify specificity.

### MIC Determinations

To quantify the susceptibility to rifampicin, a broth microdilution assay using resazurin dye was carried out (Agrawal et al., 2015). Hundred microliter of twice the required concentration of rifampicin was serial diluted across 96-well microtiter plates. Cultures were grown to exponential phase (OD_600_ 0.4–0.6), diluted to an OD_600_ of 0.1 and 100 μl was added to each well. This represents ~10^6^ cells. 2–200 ng/ml of aTc for CRISPRi/dCas9-mediated repression of *arr* gene expression was added where required. Plates were incubated at 37 °C for 40 h. Following incubation, 30 μl of 0.2 mg/ml resazurin was added to each well and incubated at 37 °C for an additional 24 h and fluorescence was measured using a Tecan microplate reader at excitation 560 nm and emission 590 nm. Images of the plates were also taken.

## Results

### CRISPRi Targeting of the Promoter Region of the *arr* Gene in *M. smegmatis* Increases the Susceptibility to Rifampicin

**Figure 1:**
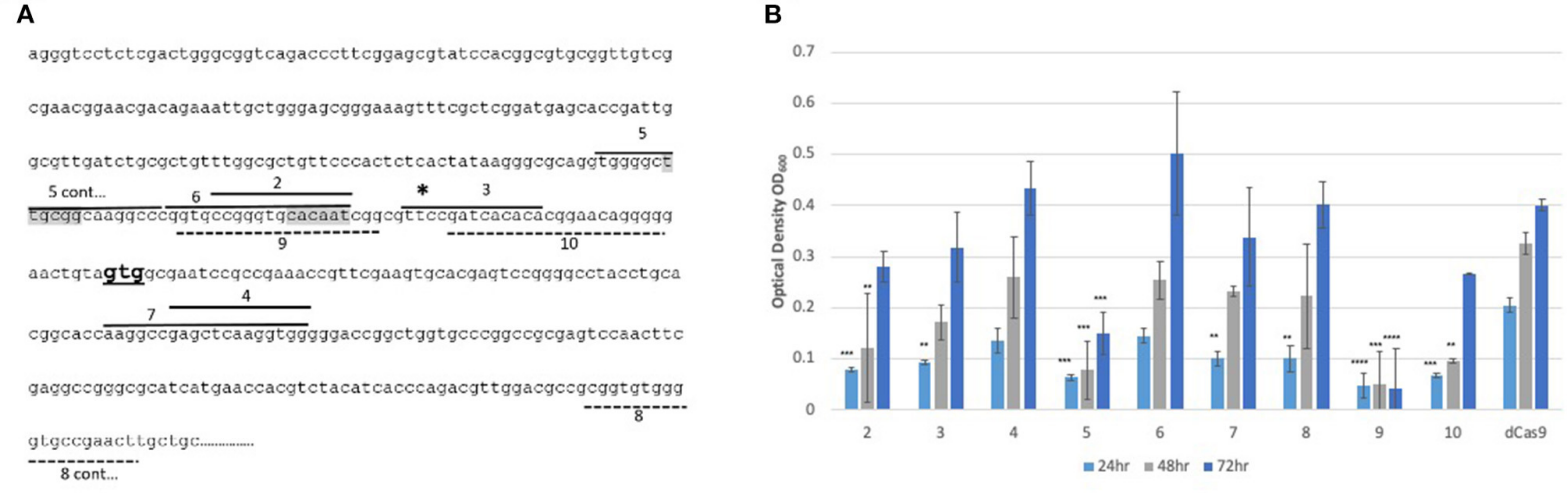
CRISPRi targeting of the *arr* gene (*MSMEG_1221*) over various sites in the promoter results in increased susceptibility to rifampicin **(A)** Position of the sgRNA targets. Nine sgRNAs (2-10) were designed to target both non-coding (sgRNAs 2-7) and coding strands (8-10). sgRNAs targeting the coding strand are indicated by a dashed line. The gene specific region has mainly 19 bp, except for sgRNAs 2 and 3 where a 13 bp region was targeted. The transcriptional start site is indicated by a * and the translation start **GTG** is in bold. Putative −10 and −35 promoter elements are highlighted in gray. **(B)** Growth of *M. smegmatis* sgRNA-dCas9 strains in 7H9 growth medium supplemented with aTc (200 ng/ml) and rifampicin (3 μg/ml). Optical density (OD_600_) was measured at 24, 48, and 72 h. Numbers refer to the sgRNA oligos as listed in Table 2. Statistical significance was determined using One-way ANOVA with Dunnett's multiple comparisons test against the dCas9-only strain for each time point (****p < 0.0001, ***p < 0.001, **p < 0.01). Error bars represent standard deviation from the mean of triplicate repeats.

Inhibition of the expression of *arr* is expected to potentiate growth inhibition in the presence of rifampicin. In order to determine which region should be targeted for CRISPRi, nine sgRNAs of different lengths were designed, targeting regions in both the promoter and coding sequences of the gene, on either the coding or non-coding strand (Figure 1A). Unlike other bacteria, mycobacteria use more commonly leaderless transcripts during gene expression, however, the *arr* gene has been shown to contain a 5′ UTR indicating the presence of a putative Shine-Dalgarno sequence (Cortes et al., 2013; Shell et al., 2015; Martini et al., 2019). Previous RNAseq studies have shown that transcription starts 31 bp upstream of the GTG annotated translational start codon (Shell et al., 2015). Putative −35 and −10 regions were predicted using Softberry BPROM (Solovyev and Salamov, 2011) and sgRNAs were designed to include coverage of these regions. The location of the sgRNAs, alongside the transcription and translational start sites and putative promoter elements is given in Figure 1A. Growth inhibition in the presence of rifampicin after induction of the various sgRNAs is shown in Figure 1B.

At 24 h, all *M. smegmatis* strains containing an sgRNA targeting *arr*, apart from those targeting regions 4 and 6, exhibited increased susceptibility (*p* < 0.05) to rifampicin compared to the control strain expressing only dCas9_Spy_. At 48 h, *M. smegmatis* strains containing sgRNAs targeting regions 2, 5, 9, and 10 still displayed significantly increased susceptibility and this endured for a further 24 h for regions 5 and 9. The shorter sgRNAs which were 13 bp long, targeting regions 2 and 3 were less effective than the 19 bp long sgRNAs. sgRNAs targeting region 5 and 9, at the promoter region were the most effective, with targeting of the −10 (sgRNA 9) region being the most impactful.

### CRISPRi Targeting of *arr* in the Absence of Rifampicin Results in Reduction of Gene Expression but Does Not Impact Growth

**Figure 2:**
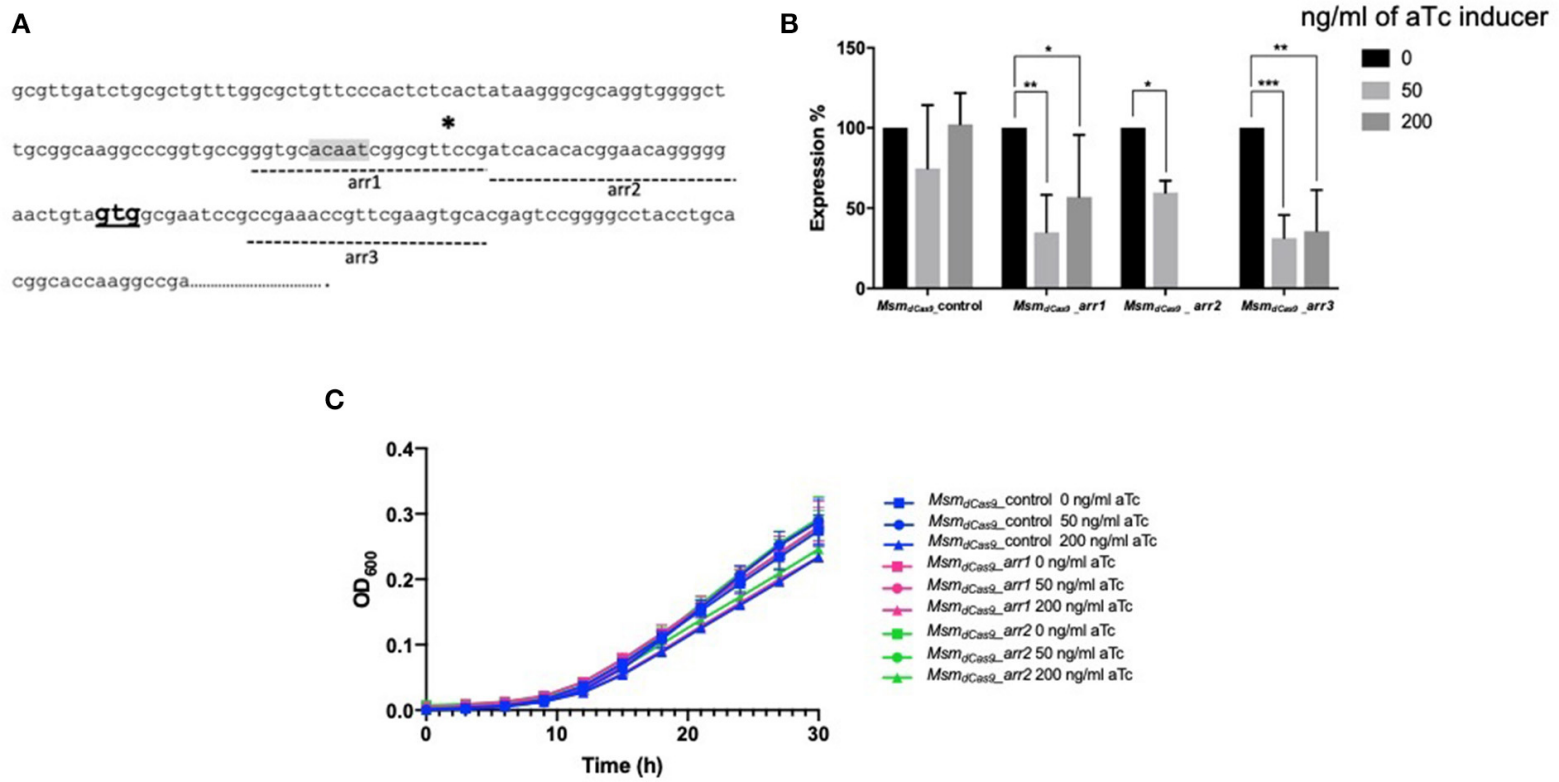
CRISPRi targeting of the non-essential gene *arr* (*MSMEG_1221*) in *M. smegmatis* results in transcriptional inhibition but does not impact growth. **(A)** Position of sgRNA targets on the *arr* gene. Three sgRNAs were designed to target *arr*, indicated by dashed lines. *arr1* targeted −47 to −28, *arr2* targeted −27 to −8 bp upstream of the annotated translational start codon. *arr3* targeted +13 to +32 bp within the coding sequence. The transcriptional start site is indicated by a * and the translation start site **(GTG)** is in bold. The putative−10 promoter element predicted using Softberry BPROM is highlighted in gray. **(B)** Inhibition of *arr* expression in cultures expressing sgRNA targeting *arr*, induced with different concentration of aTc. mRNA levels are expressed relative to the control strain expressing dCas9 only. Results are the mean of three independent biological replicates and expression levels were measured in duplicate for each concentration of aTc (*p < 0.05, **p < 0.01, ***p < 0.001); **(C)** Growth of *M. smegmatis* expressing either sgRNA targeting *arr* or dCas9 only with differing levels of aTc. Cultures were grown in 96 well plates from a starting OD_600_ in a total volume of 200 μl.

Three further sgRNAs were designed (arr1, arr2, and arr3) that targeted a 20 bp gene specific region designed to cover the putative −10 region (arr1), the 5′ UTR (arr2) and the 5′ end of the coding sequence (arr3) (Figure 2A). All sgRNAs were designed to target the coding strand. The impact of induction of sgRNAs arr1, arr2 and arr3 alongside dCas9 on the expression of the *arr* gene was measured using RT-qPCR. Expression levels were normalized to *sigA* and expressed relative to the uninduced cultures. The results are shown in Figure 2B. Induction of arr1 and arr3 with aTc at 50 ng/ml led to more than 50% reduction in the expression of the *arr* gene. Induction of arr2 with 50 ng/ml of aTc resulted in 40% reduction in its expression. Induction with higher levels of aTc (arr1, arr3 with 200 ng/ml) did not improve inhibition of *arr* expression.

As *arr* is a non-essential gene, inhibition of its expression is not expected to influence the growth of the bacteria. To test this, we compared the effect of the induction with aTc on the growth of *Msm*_*dCas*9_*_arr1* and *Msm*_*dCas*9_*_arr2*. Additionally, we compared the impact of CRISPRi induction on the growth of the *Msm*_*dCas*9_*_control* strain which does not contain a targeting sgRNA. The results (Figure 2C) show that there is no growth inhibitory effect as a result of induction of the CRISPRi system

**Figure 3:**
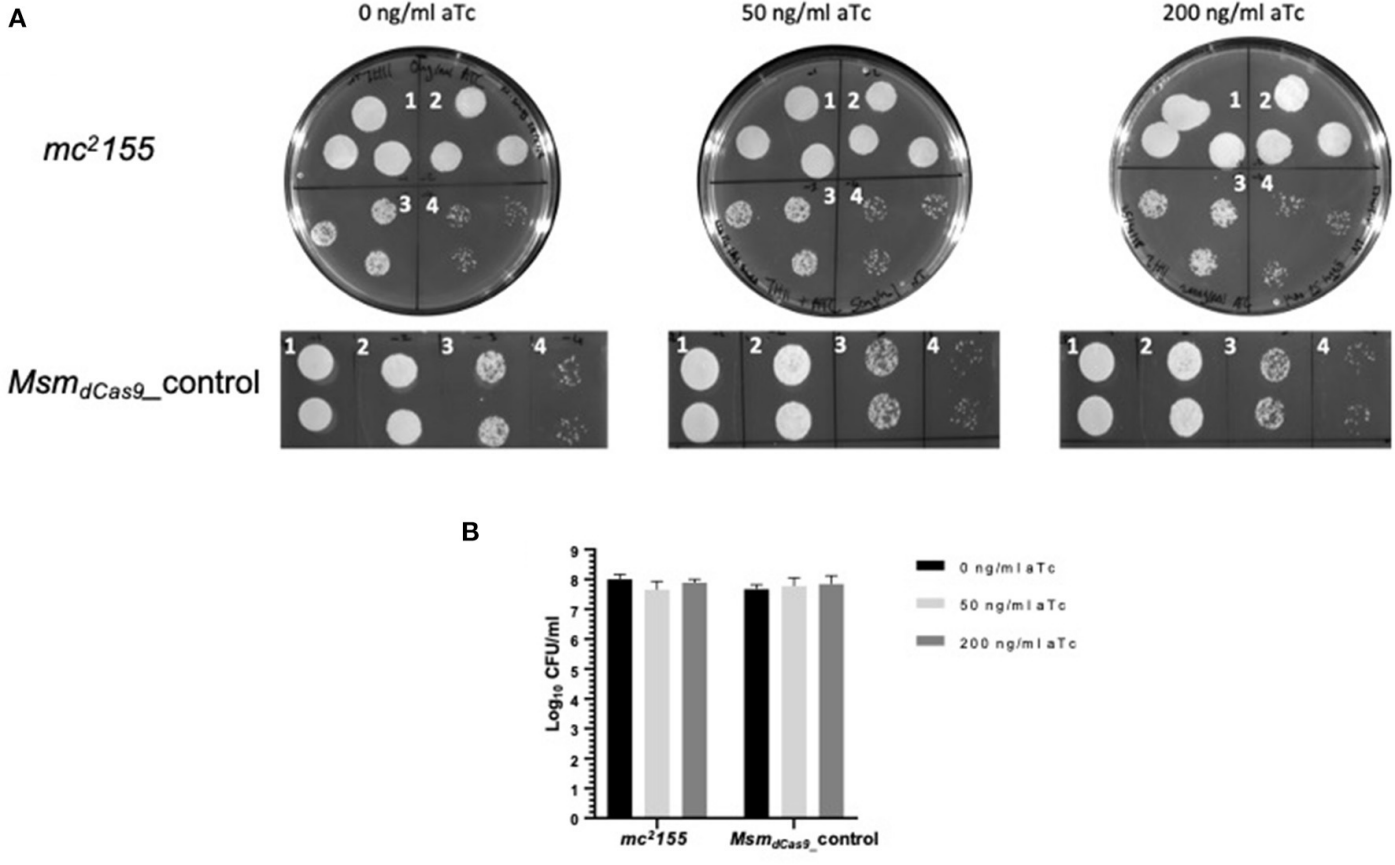
Induction of dCas9_Spy_ in *M. smegmatis* mc^2^155 is not toxic to the cells. **(A)** Miles and Misra spotting to examine the impact of induction of dCas9 on the viability of *M. smegmatis* mc^2^155. Broth cultures of Msm_WT and Msm_dCas9__control strains were grown overnight at 37 °C and diluted to OD_600_ 0.01. Cultures were further diluted: 1- 10^−1^, 2- 10^−2^, 3- 10^−3^, 4- 10^−4^ as labeled, and spotted using Miles and Misra on 7H11 agar supplemented with 0, 50, and 200 ng/ml of aTc. **(B)** The CFU/ml was estimated from colony counts.

Initial studies using dCas9 from *S. pyogenes* to repress expression of a number of essential genes reported no toxicity in mycobacteria (Choudhary et al., 2015; Singh et al., 2016). However, a subsequent study reported toxicity (Rock et al., 2017). Given the discrepancies over the reports of toxicity in the literature, the effect of induction of dCas9_Spy_ on the viability of *M. smegmatis* mc^2^155 was evaluated. *M. smegmatis* cultures expressing dCas9_Spy_ (*Msm*_*dCas*9_*_control*) were induced with 50 and 200 ng/ml of aTc and compared to non-induced cultures and the wild type strain. The results, which are shown in Figure 3, indicate that induction of with aTc does not result in toxicity.

### CRISPRi Targeting *arr* Sensitizes *M. smegmatis* to Rifampicin

An initial qualitative screen on solid media demonstrated that silencing of *arr* by induction of the sgRNAs with a range of aTc concentrations (50-200 ng/ml) sensitized *M. smegmatis* to rifampicin (Supplementary Figure 1). In order to quantify the effect of *arr* silencing on rifampicin sensitivity, the MIC of rifampicin for *Msm*_*dCas*9_*_arr1, Msm*_*dCas*9_*_arr2* and *Msm*_*dCas*9_*_arr3* was determined without aTc and with aTc at 50 ng/ml and 200 ng/ml (Table 3, Supplementary Figure 2). Induction of the CRISPRi system using aTc had no impact on the MIC of the wild type strain, mc^2^155. The MIC obtained (1.6–3.12 μg/ml) is in-line with previously reported values [0.8–3.9 μg/ml (Agrawal et al., 2015; Gupta et al., 2015)]. The non-induced cultures and the *Msm*_*dCas*9_*_control* showed the same MIC as the wild type strain, *mc*^2^*155* (Supplementary Figure 2 wells 2-5). However, at 50 ng/ml and 200 ng/ml aTc there was a clear decrease in the MIC of rifampicin for the three strains (*Msm*_*dCas*9_*_arr1, Msm*_*dCas*9_*_arr2* and *Msm*_*dCas*9_*__arr3*) to 0.2 μg/ml with 50 ng/ml of aTc and 0.8 μg/ml with 200 ng/ml of aTc. The reduction in MIC due to the induction of the sgRNAs with 50 ng/ml and 200 ng/ml of aTc were 8–16 and 2–4-fold, respectively (Table 3). Deletion of the *arr* gene in *M. smegmatis* has been reported to decrease the MIC of rifampicin by 16-fold, similar to induction with 50 ng/ml of aTc (Agrawal et al., 2015).

**Table 3:** Fold change in the MIC due to inhibition of *arr* expression.

**Strains**	**MIC of rifampicin (μg/ml)**			**MIC fold change**	
	**0 ng/ml aTc**	**50 ng/ml aTc**	**200 ng/ml aTc**	**50 ng/ml aTc**	**200 ng/ml aTc**
mc^2^155	1.6–3.12	1.6–3.12	1.6–3.12	-	-
Msm_dCas9__arr1	1.6–3.12	0.2	0.8	8–16	2–4
Msm_dCas9__arr2	1.6–3.12	0.2	0.8	8–16	2–4
Msm_dCas9__arr3	1.6–3.12	0.2	0.8	8–16	2–4

### Lower Levels of aTc Can Be Used to Sensitize the Strains to Rifampicin

**Figure 4:**
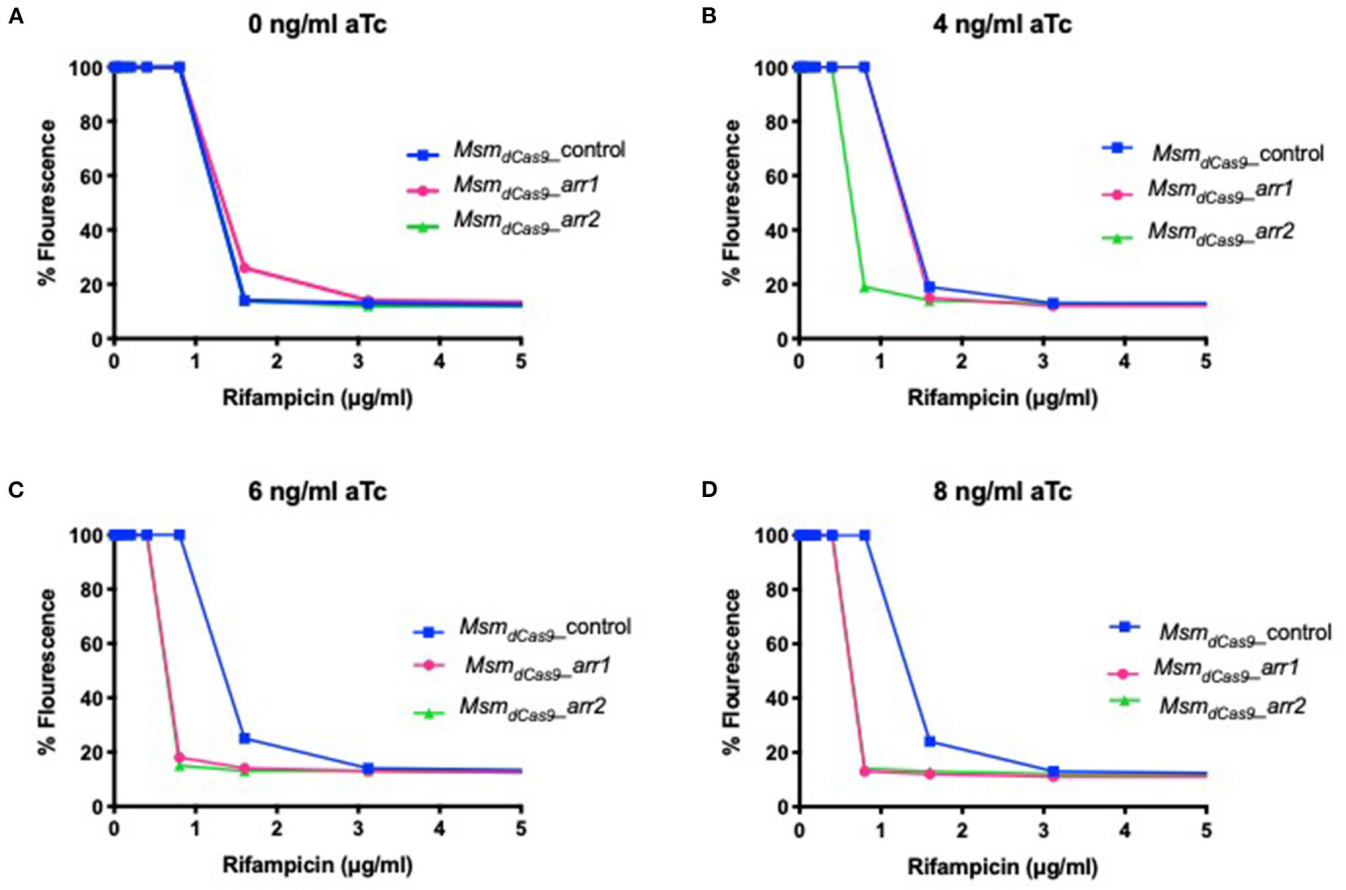
Induction of dCas9_Spy_ with low concentrations of aTc sensitizes *Msm*_*dCas*9_*_arr1* and *Msm*_*dCas*9_*_arr2* to rifampicin. 96 well microtiter plates were filled with 100 μl of twice the required concentration of rifampicin and serial diluted in two-fold across the plates. Approximately 10^6^ bacterial cells were added to each well with antibiotics containing **(A)** 0 ng/ml **(B)** 4 ng/ml (**C)** 6 ng/ml and **(D)** 8 ng/ml of aTc. Plates were incubated at 37°C for 40 h after which 30 μl of 0.2 mg/ml resazurin were added to each well and incubated at 37°C for an additional 24 h. Fluorescence was measured on a Tecan plate reader at 560 nm (excitation) and 590 nm (emission).

To investigate whether sensitization to rifampicin could be achieved using lower concentrations of aTc, the MIC of rifampicin for the strains *Msm*_*dCas*9_*_control, Msm*_*dCas*9_*_arr1* and *Msm*_*dCas*9_*_arr2* were measured in the presence of 4 ng/ml, 6 ng/ml and 8 ng/ml of aTc and compared to non-induced cultures. The results, which are displayed as a change in fluorescence at different concentrations of rifampicin, show that without induction, all the three strains have the same MIC (1.6 μg/ml, Figure 4). However, a 2-fold reduction in the MIC is achieved at 4, 6 and 8 ng/ml of aTc. This suggests that silencing of *arr* by induction of the sgRNA can take place at concentrations as low as 4 ng/ml, although the impact on the level of sensitization is less than that observed at higher concentrations.

## Discussion

In this study, we demonstrate the usefulness of CRISPRi silencing for the study of genes that are not essential for growth, but encode targets associated with antibiotic resistance. Induction of dCas9_Spy_ in *arr* targeting strains with 50 and 200 ng/ml of aTc decreased the MIC of rifampicin and re-sensitized *M. smegmatis* to the antibiotic by 8-16 and 2-4-fold, respectively. *M. smegmatis*, which is intrinsically resistant to rifampicin due to the expression of *arr*, has previously been shown to be a useful surrogate for pathogenic mycobacteria to study antimicrobial susceptibility (Chaturvedi et al., 2007). Additionally, as rifampicin is used for the treatment of several mycobacterial infections, *arr* seemed to be a suitable target to examine the potential use of the CRISPRi/dCas9 system to explore genes which could be targeted to improve the efficacy of current antibiotics. This approach can now be used to study genes in other intrinsically rifampicin-resistant species such as *Mycobacterium abscessus*, a pathogenic fast growing mycobacterial species. Interestingly, *M. abscessus* also carries a putative *arr* gene and its role in intrinsic rifampicin resistance was previously demonstrated through deletion of *arr* (Rominski et al., 2017). The work presented here demonstrates that gene silencing can be used effectively in mycobacterial species to study intrinsic drug resistance.

In this study we show that CRISPRi/dCas9 can be used to restore sensitivity to rifampicin in *M. smegmatis* by silencing the expression of *arr*. As little as 40–60% reduction in expression of *arr* resulted in an 8–16-fold decrease in the MIC. Previous studies using an Δ*arr* strain reported a 16-fold decrease in the MIC (Agrawal et al., 2015). This suggests that, for *arr* at least, silencing can be as effective as deletion as the levels of susceptibility reached were similar to that previously reported. Although the Δ*arr* strain from the Agrawal study was not included here, the congruity of the MIC measurements in the wild type strains between this study and the study by Agrawal et al. validates our approach. To our knowledge there are no other isogenic Δ*arr* mutants to which a comparison could be made. Although, another Δ*arr* strain of *M. smegmatis* has been described, this is in a different genetic background, therefore not comparable to this study (Combrink et al., 2007).

Similar to previous reports (Larson et al., 2013), the findings of this study show that gene silencing using dCas9_Spy_ is most effective if the target site is within the promoter region on the coding or non-coding strand of the gene of interest inhibiting transcription initiation. Targeting regions 5 and 9, both of which were within the promoter region, on the non-coding and coding strand respectively, was most impactful in comparison to the other target regions (Figure 1B). Additionally, targeting region 9, which include the putative −10 sequence, produced the most sustained impact on growth 24, 48, and 72 h post-induction. Strategies to inhibit the elongation of transcription (rather than transcription initiation) are reported to be most effective when targeting the coding strand of the protein coding region or the 5′ UTR (Larson et al., 2013). These are represented by sgRNAs 10 and 8 (coding) with sgRNAs 4 and 7 targeting the non-coding strand. Of those sgRNAs targeting sites beyond the transcription start site, sgRNA 10 was the most effective in improving the susceptibility to rifampicin thereby supporting the strategy of targeting the coding strand. The results further showed that the shorter sgRNAs (sgRNAs 2, 3, and 4) were less effective than longer sequences.

The three main sgRNAs used in this study were designed to target the putative −10 region (arr1), the 5′ UTR (arr2) and the 5′ end of the coding sequence (arr3), with all three being 20 bp long. The impact of induction of the system for 1 h with 0, 50 and 200 ng/ml of aTc was measured using RT-qPCR. *arr* expression was suppressed by 40–60% (Figure 2B). Previous studies have measured levels of silencing by CRISPRi/dCas9_Spy_ 12–24 h post-induction with aTc. Singh et al. (2016) reported a 60–80% reduction in *sigH* and *pknB* expression following induction with 200 ng/ml of aTc for 12 h (Singh et al., 2016). This study shows that gene silencing can take place within 1 h of induction. Additionally, the data presented here show that 200 ng/ml of aTc did not improve gene silencing and sensitization compared to 50 ng/ml (Figure 2B and Table 3). In *M. smegmatis*, the TetR-regulated UVtetO promoter has been reported to achieve the peak of induction with 50–100 ng/ml of aTc with decreasing levels of induction at concentrations higher than 100 ng/ml (Ehrt et al., 2005) and this could explain our observations at 200 ng/ml. Furthermore, this study also shows that induction with lower concentrations (4–8 ng/ml) of aTc is sufficient to decrease the MIC of rifampicin (Figure 4). One study indicated that dCas9_Spy_ expression is potentially toxic in mycobacteria (Rock et al., 2017). Toxicity of dCas9_Spy_ has also been reported in *E. coli* upon very high levels of expression (Cho et al., 2018). However, similar to other reports (Choudhary et al., 2015, 2019; Singh et al., 2016), the work presented here shows that the induction of dCas9_Spy_ does not impact *M. smegmatis* growth and viability.

These results show that the CRISPRi system can be used to target, and knock-down genes involved in antimicrobial resistance, enabling faster reverse genetics compared to gene knockout techniques. There are limitations, given the requirement for induction by aTc, however, the system has great potential for multiplexing which will be useful in the study of redundancy. In particular, it will be useful in the study of multiple drug efflux pumps in slow-growing pathogenic mycobacterial genomes where sequential mutagenesis is not efficient. The approach can improve studies on gene function and resistance to current and future antimicrobials.

## Data Availability Statement

The raw data supporting the conclusions from this article will be made available by the authors on request. The raw data used to derive the MICs in Table 3 is given in Supplementary Figure 2.

## Author Contributions

VF, SG, SW, and SLK designed the study and analyzed the data. VF, AAC, SG, AB, AJG, and OL carried out the experiments. SLK and BWW did funding acquisition. VF and SLK wrote the first draft of the manuscript. All authors contributed to the manuscript revision, read, and approved the submitted version.

## Conflict of Interest

The authors declare that the research was conducted in the absence of any commercial or financial relationships that could be construed as a potential conflict of interest.

## Supplementary Material

Supplementary Figure 1 CRISPRi targeting of *arr* sensitizes *M. smegmatis* to rifampicin. The bacterial cultures were grown to saturation for 48 h in 7H9 and streaked on 7H11 agar plates containing different concentrations of aTc (50 ng/ml-200 ng/ml) as indicated. Plates contained rifampicin at 2 μg/ml. Plates were incubated at 37°C for 48 h before being photographed.

Supplementary Figure 2 Induction of dCas9_Spy_ in *arr* targeting strains decreases the MIC of rifampicin. Ninety-Six well microtiter plates were filled with 100 μl of twice the required concentration of rifampicin and serially diluted in 2-fold across the plates. Approximately 10^6^ bacterial cells were added to wells with antibiotics and **(A)** 0 ng/ml **(B)** 50 ng/ml (**C)** 200 ng/ml of aTc. Plates were incubated at 37°C for 40 h after which 30 μl of 0.2 mg/ml resazurin was added to each well and incubated at 37°C for an additional 24 h. Fluorescence was measured on a Tecan plate reader at 560 nm (excitation) and 590 nm (emission) and images taken. Pink wells indicate bacterial growth whereas blue wells indicate inhibition of growth in the presence of rifampicin. Images shown are representative of one replicate for each treatment. The MIC is determined as the concentration of rifampicin at which the first well in the row appears pink.
