# Supplementary figures and images for: Re-sensitization of Mycobacterium smegmatis to Rifampicin Using CRISPR Interference Demonstrates Its Utility for the Study of Non-essential Drug Resistance Traits
Source: Front Microbiol. 2021 Feb 1;11:619427. doi: 10.3389/fmicb.2020.619427 (PMC7882622; doi:10.3389/fmicb.2020.619427)

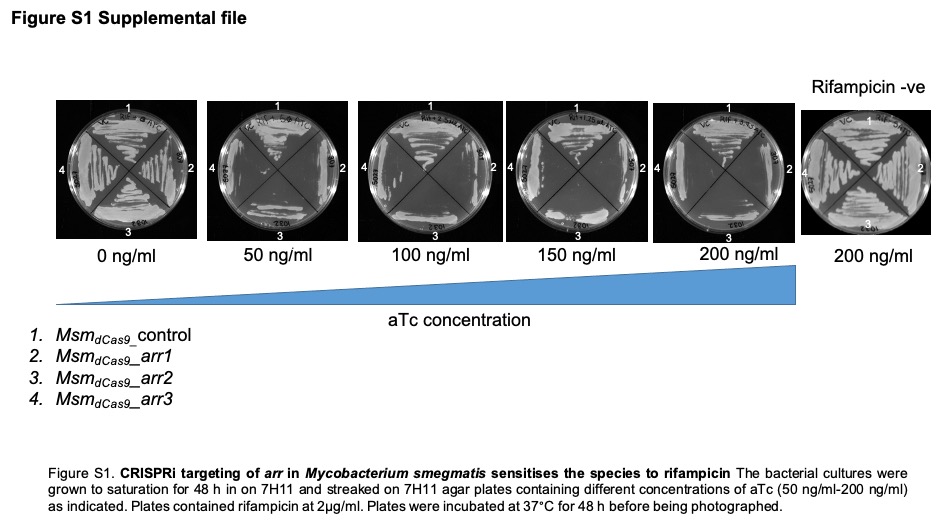

Supplement: Supplementary Figure 1 — CRISPRi targeting of arr sensitizes M. smegmatis to rifampicin. The bacterial cultures were grown to saturation for 48 h in 7H9 and streaked on 7H11 agar plates containing different concentrations of aTc (50 ng/ml-200 ng/ml) as indicated. Plates contained rifampicin at 2 μg/ml. Plates were incubated at 37°C for 48 h before being photographed. [file Image_1.JPEG]

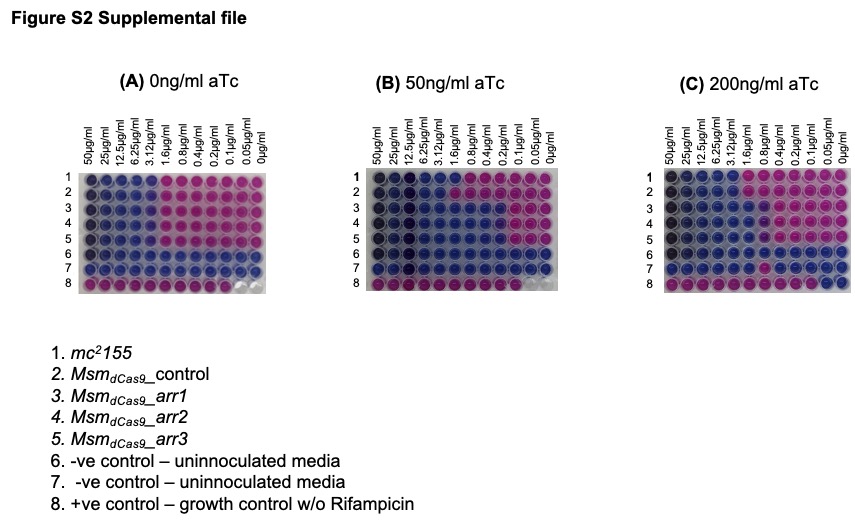

Supplement: Supplementary Figure 2 — Induction of dCas9Spy in arr targeting strains decreases the MIC of rifampicin. Ninety-Six well microtiter plates were filled with 100 μl of twice the required concentration of rifampicin and serially diluted in 2-fold across the plates. Approximately 106 bacterial cells were added to wells with antibiotics and (A) 0 ng/ml (B) 50 ng/ml (C) 200 ng/ml of aTc. Plates were incubated at 37°C for 40 h after which 30 μl of 0.2 mg/ml resazurin was added to each well and incubated at 37°C for an additional 24 h. Fluorescence was measured on a Tecan plate reader at 560 nm (excitation) and 590 nm (emission) and images taken. Pink wells indicate bacterial growth whereas blue wells indicate inhibition of growth in the presence of rifampicin. Images shown are representative of one replicate for each treatment. The MIC is determined as the concentration of rifampicin at which the first well in the row appears pink. [file Image_2.JPEG]
